# Isolated gastric Langerhans cell histiocytosis: a case report and literature review

**DOI:** 10.3389/fonc.2026.1724994

**Published:** 2026-08-25

**Authors:** Xiaohan Ning, Yuan Tao, Yulu Wang, Kang Wan, Ling Wang, Qi Sun, Shuhua Yi, Shuhui Deng

**Affiliations:** 1 Department of Hematopathology, Qingdao Central Hospital, University of Health and Rehabilitation Sciences (Qingdao Central Hospital), Qingdao, Shandong, China; 2 State Key Laboratory of Experimental Hematology, National Clinical Research Center for Blood Diseases, Haihe Laboratory of Cell Ecosystem, Institute of Hematology & Blood Diseases Hospital, Chinese Academy of Medical Sciences & Peking Union Medical College, Tianjin, China; 3 Jiangxi Provincial Key Laboratory of Hematological Diseases, Department of Hematology, The First Affiliated Hospital, Jiangxi Medical College, Nanchang University, Nanchang, Fujian, China

**Keywords:** adult, BRAF V600E, isolated gastric, Langerhans cell histiocytosis, Langerhans cells

## Abstract

Langerhans cell histiocytosis (LCH) is a rare histiocytic disease that predominantly affects young children, with adult cases being exceedingly uncommon. The most frequently affected sites in LCH are bones, lungs, and the pituitary gland, in whom gastrointestinal involvement is scarce. Primary isolated gastric LCH is rarely seen and typically manifests as a polypoid lesion. This article presented a case of LCH in an adult patient, while endoscopic examination revealed an erosive lesion on the greater curvature of the gastric antrum, confirmed by immunohistochemistry. We further discuss the pathogenesis, endoscopic and histopathological characteristics, immunohistochemical findings, relevant differential diagnoses, and current treatment strategies. This report aims to raise clinicians’ awareness of the various endoscopic presentations for LCH and emphasizes the importance of thorough histopathological evaluation to obtain an accurate diagnosis and avoid oversight or misdiagnosis.

## 1 Introduction

LCH is a malignant histiocytic disease derived from myeloid dendritic cells (1), classified as a rare disease. The incidence rate in children is approximately 3–5 per million, while in adults, it is 1–2 per million (2). The male-to-female ratio is 2.1:1. Although LCH can affect multiple organs or systems throughout the body, gastrointestinal involvement is rare in adults (3, 4), accounting for only about 2% in all LCH patients. Gastrointestinal LCH most frequently involves the colon and rectum, which typically presents as a single colorectal polyp (5, 6). Isolated gastric LCH is seldom seen. Most affected patients are either asymptomatic or only mild gastrointestinal symptoms. Under endoscopy, most cases manifest as solitary polyps or submucosal protruding lesions, while fewer cases present with erosive or ulcerative changes. They are prone to misdiagnosis or missed diagnosis due to their non-specific manifestations (7). We reported an adult case of isolated gastric LCH presenting with an erosive lesion in the gastric antral mucosa and reviewed the relevant literature on isolated gastric LCH to enhance clinicians’ understanding about this disease.

## 2 Clinical data

### 2.1 Previous medical history

A 37-year-old male was admitted due to “Upper abdominal distension for four years and diarrhea for one year”. Four years ago, the patient experienced epigastric distension without obvious causes, accompanied by acid reflux and hiccups. The patient denied cough, dyspnea, and chest pain, as well as polyuria, nocturia, and cognitive impairment. An external gastroscopy examination suggested atrophic gastritis. Although the patient received acid-suppressing and stomach-protecting drugs, his symptoms showed no significant improvement. One year ago, he developed intermittent diarrhea, with pasty stools occurring 3–4 times per day. One month before admission, follow-up gastroscopy and colonoscopy were performed at local hospital. There were no abnormalities in colonoscopy, while gastroscopy showed one 0.5 cm × 0.5 cm erosion of the anterior wall mucosa on the greater curvature side of the gastric antrum. A preliminary diagnosis suggested chronic atrophic gastritis with erosion. A biopsy was obtained from the greater curvature mucosa of the gastric antrum. Pathological examination showed diffuse heterogeneous cells within the local mucosa, which were consistent with a malignant tumor and prone to a low-adhesive carcinoma. It was recommended to perform immunohistochemistry for further diagnosis.

### 2.2 Current hospitalization data

**Figure 1:**
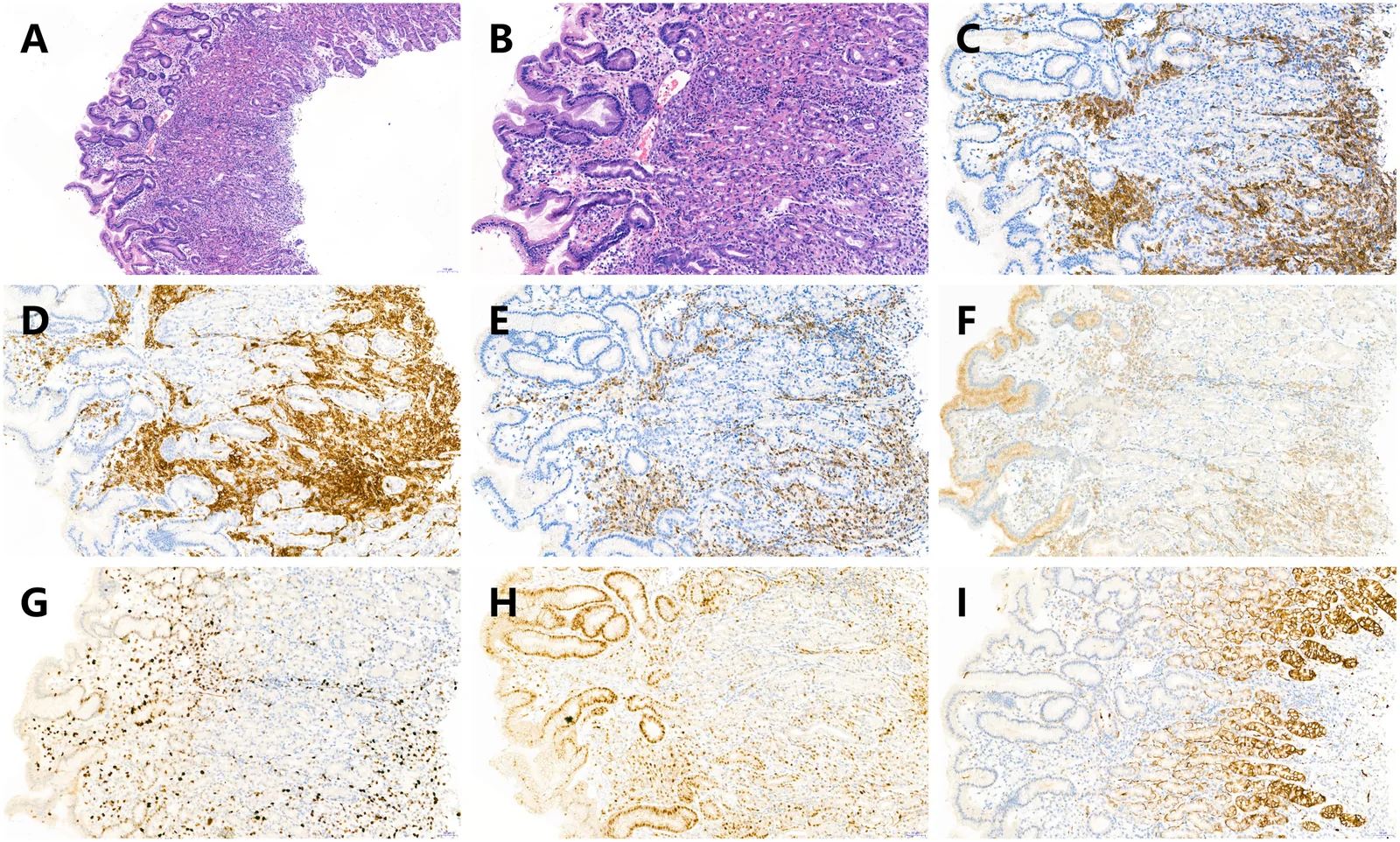
Morphological characteristics and immunohistochemical staining results. **(A)** Under low magnification, Langerhans cells with a small number of lymphocytes and eosinophils are observed in the background (HE, ×100). **(B)** Under high magnification, the nuclei of Langerhans cells exhibit oval or slightly irregular morphology. Some of the nuclei show nuclear grooves and thin nuclear chromatin (HE, ×200). The atypical cells show uniform positivity for CD1a [**(C)**, ×200], Langerin [**(D)**, ×200], S100 [**(E)**, ×200], and BRAF V600E [**(F)**, ×200]. Ki67 positive rate is approximately 30% [**(G)**, ×200]. Cyclin D1 shows nuclear positivity in the minority of the atypical cells [**(H)**, ×200]. CD56 is negative [**(I)**, ×200].

**Figure 2:**
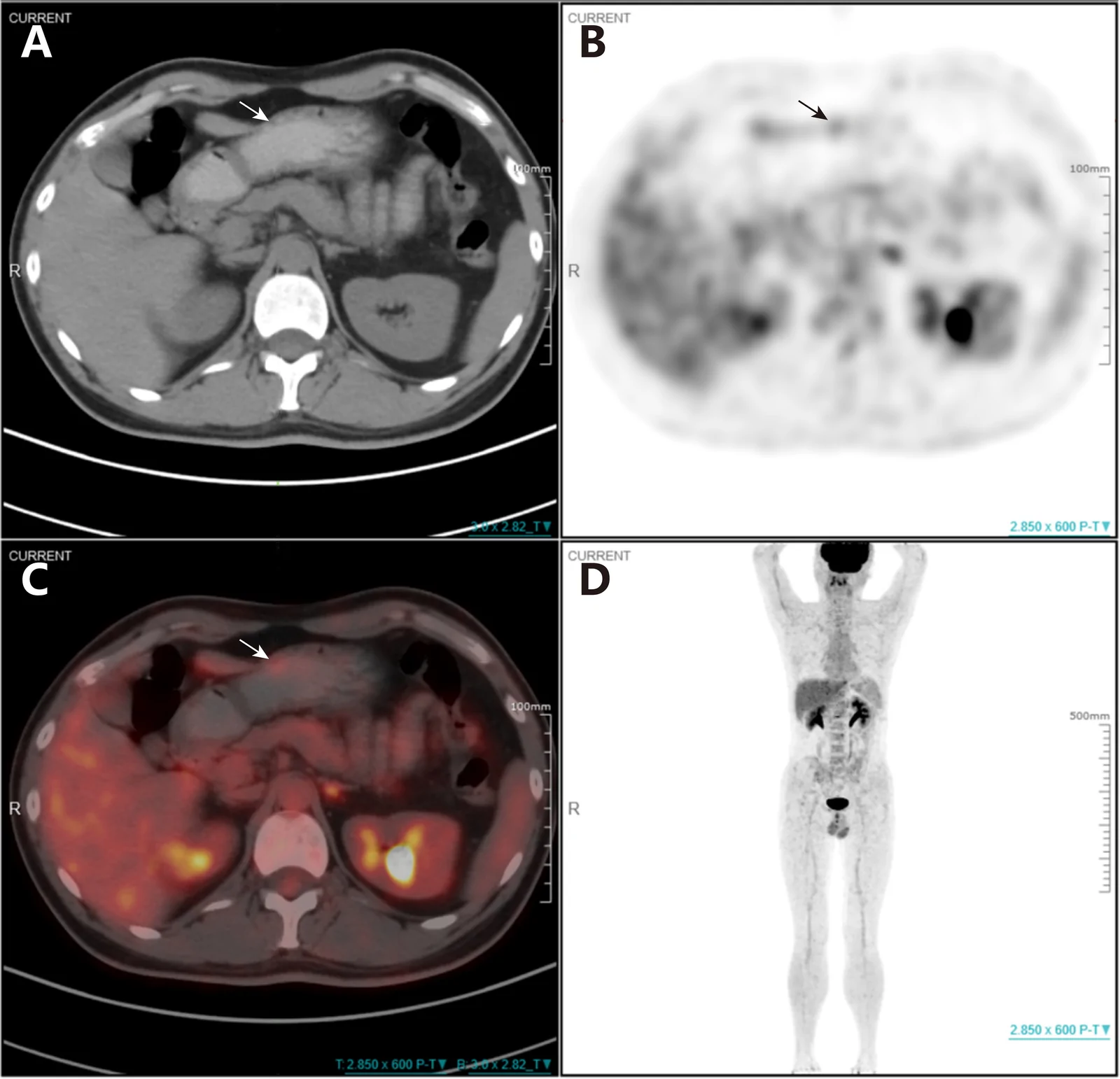
An abdominal CT scan is shown in Image **(A)**, with the stomach indicated by a white arrow. Image **(B)** presents a PET scan, in which a gastric lesion with focally increased nodular FDG uptake is marked by a black arrow. The fused PET/CT image in Image **(C)** highlights increased metabolic activity in the anterior wall of the gastric antral greater curvature, with a maximum standardized uptake value (SUVmax) of approximately 2.7, as indicated by a white arrow. Finally, a maximum intensity projection (MIP) image from the whole-body PET scan is displayed in Image **(D)**, which shows no evidence of lymphoma or other tumors.

To further clarify the diagnosis, the patient was evaluated at the Institute of Hematology & Blood Diseases Hospital, Chinese Academy of Medical Sciences on December 11, 2024. His medical history was the same as above described, with no significant personal or family history. Physical examination revealed no generalized skin rash, no tremor of the extremities, steady gait without ataxia, and normal muscle tone. Physical examination showed no abnormalities. Blood tests (including complete blood count, liver and kidney functions, electrolytes, lactate dehydrogenase, autoantibodies, and endocrine assessment such as pituitary function and serum osmolality) were all within normal limits. Bone marrow puncture and biopsy showed no significant abnormalities. Pathological consultation of the gastric mucosal lesion from the external hospital demonstrated the infiltration of atypical cells among the glandular structures in the mucosal layer. These nuclei exhibited oval or slightly irregular morphology. Some of the nuclei showed nuclear grooves and thin nuclear chromatin. There were also a small number of lymphocytes and eosinophils presenting in the background (**Figures 1A, B**). Immunohistochemical staining results were as follows: Langerin+, CD1a+, S100+, BRAF V600E+, Vimentin+, CD68+, CD4+, a small amount of cyclin D1 positivity, and Ki67 positive rate approximately 30% (**Figures 1C–H**). Negative markers included: Desmin-, SOX10-, CD56- (**Figure 1I**), HMB45-, CD79a-, CK-, CD19-, MUM1-, CD138-, P40-, CD38-, CD20-, SYN-, SMA-, and PAX5-. Conclusion: Langerhans cell histiocytosis (involving the anterior wall of the greater curvature of the gastric antrum). No abnormalities were identified on cranial CT, chest CT, abdominal ultrasound, and echocardiography. Fluorodeoxyglucose-positron emission tomography-CT (FDG-PET-CT) scan showed a slightly increased metabolic activity (SUVmax ≈ 2.7) on the anterior wall of the greater curvature of the gastric antrum. The arrow pointed to the location of the lesion (**Figure 2**). No abnormalities were found in other organs, and final diagnosis should be combined with gastroscopic pathological results. After systematic examination ruled out multi-systemic diseases, the final diagnosis was isolated gastric LCH.

## 3 Discussion

**Figure 3:**
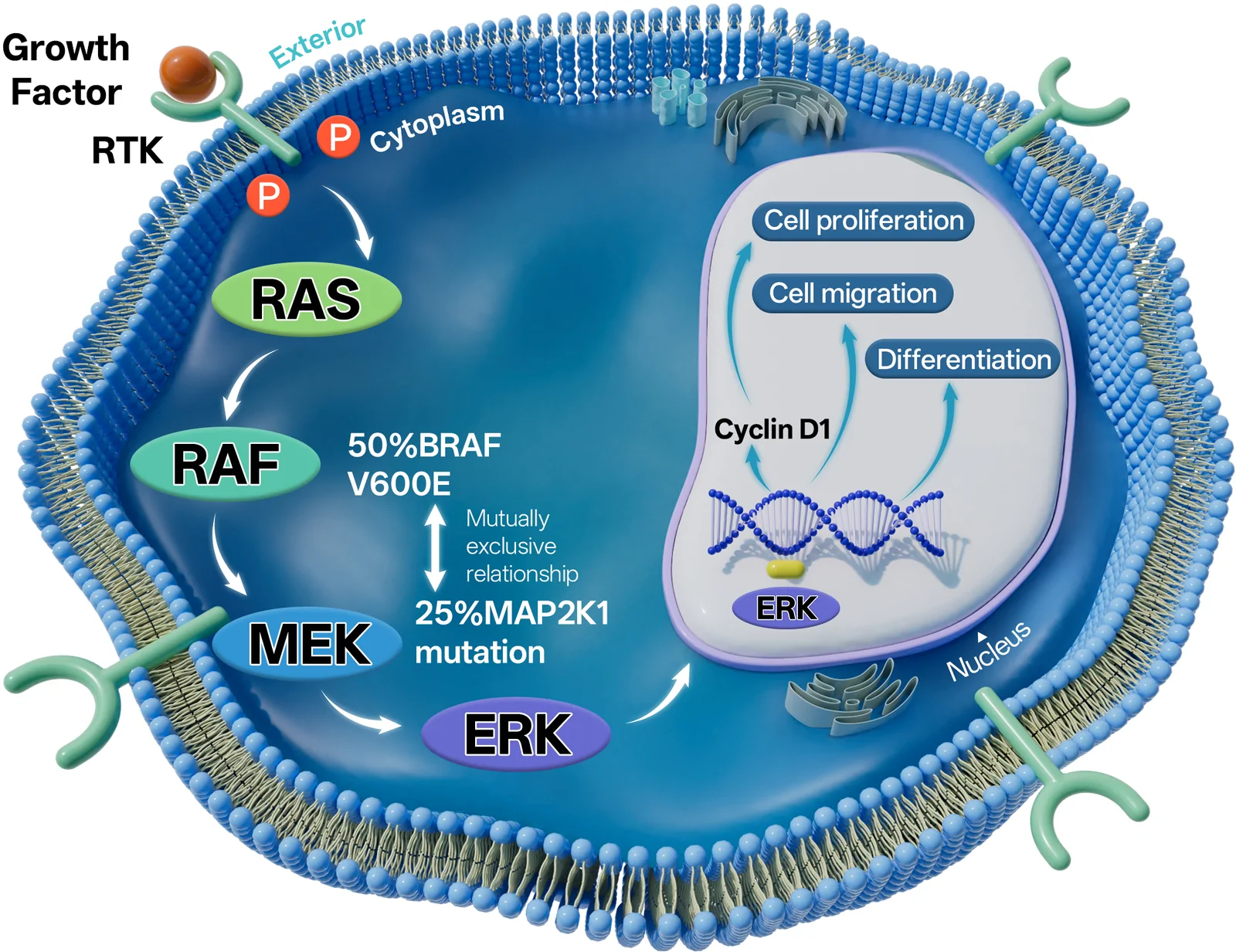
Different genetic alterations in LCH. It is currently believed to be a clonal disease caused by the activation of RAS-RAF-MEK-ERK pathway. There is a mutually exclusive relationship between BRAF V600E mutations (approximately 50%) and MAP2K1 mutations (around 25%). These two mutations can play an important role in driving the proliferation of Langerhans cells through the universal MAPK-ERK pathway in downstream.

LCH is a dendritic cell tumor caused by clonal proliferation of Langerhans cells (8). It was previously regarded as an inflammatory lesion until 2010, when recurrent somatic BRAF V600E mutations (9) were identified in the majority of LCH patients, proving its malignant nature. Regarding the pathogenesis of LCH, it is currently believed to be a clonal disease caused by the activation of RAS-RAF-MEK-ERK pathway (10). Approximately 50% patients have BRAF V600E mutations (**Figure 3**). Another 50% patients, MAP2K1 mutations are up to 50%, which are the members of mitogen-activated protein kinase (MAPK). While there is a mutually exclusive relationship between BRAF V600E mutations and MAP2K1 mutations (11, 12). Additional a few mutations have already been reported in literature, such as BRAF in-frame deletions, ARAF, NRAS, and KRAS mutations (13, 14). They both lead to a nearly universal activation of MAPK-ERK pathway in downstream, which in turn upregulates cyclin D1 expression. Researches on the prognostic significance of different mutations are also being explored.

LCH is a heterogeneous disease that can affect multiple systems throughout the body (15). The most commonly affected sites include bones, lungs, and the central nervous system (CNS). The clinical manifestations show different characteristics depending on the involved organs. Bone lesions mainly present as osteolytic changes; lung involvements may cause cough, chest pain, and dyspnea; and the pituitary gland is the most frequently affected site in the CNS, mainly leading to central diabetes insipidus. Based on different patterns of lesion distribution, LCH can be classified as single-system unifocal LCH, single-system multifocal LCH, and multi-system LCH (16). Currently, there is no standard treatments for adult LCH. Generally, single system single-site involvement can be followed up or conducts a local treatment, such as the surgical resection. For single-system multiple-lesion or multi-system involvement, systemic treatments are recommended, primarily consisting of chemotherapy or targeted agents (17). The commonly used chemotherapy drugs comprise cytarabine monotherapy, cytarabine combined with methotrexate or clatbine monotherapy, etc. Patients with BRAF V600E mutations may benefit from BRAF inhibitors, for example vemurafenib (18, 19).

The incidence rate of LCH in adults is low, and isolated gastric involvement is exceedingly rare (20). Previous reports on isolated gastric LCH are limited. A comprehensive literature analysis indicates that male patients are predominant in adult LCH (21), while female patients are more frequently affected in isolated gastric LCH (3, 22–24), suggesting potential differences in gender distribution among the different subtypes of LCH. The median age of onset for isolated gastric LCH ranges from 46.5 to 56 years (5, 22), which is consistent with the general population of adult LCH. The patient in this case was a 37-year-old male, with a relatively young age at the onset, possibly due to early detection.

In isolated gastric LCH, approximately 50% of patients may be asymptomatic (25), only detected during endoscopic examination. Some patients may present with non-specific gastrointestinal symptoms, for instance abdominal distension, abdominal pain, nausea, diarrhea, or heartburn. The most commonly affected sites of isolated gastric LCH are the gastric body, the gastric antrum, and the junction between the gastric body and gastric antrum (24). It rarely invades the entire stomach. Most patients exhibit a single lesion. The area of endoscopic lesions ranges from 0.2 cm × 0.3 cm to 1.0 cm × 1.0 cm (22). Endoscopic manifestations exhibit diverse characteristics and are categorized into submucosal protruding and non-protruding lesions. Protruding lesions primarily consist of solitary polyps or simple submucosal protrusions, while non-protruding lesions mainly contain erosive or ulcerative lesions. Nozaki et al. (26) reported a case of a middle-aged man that a red lesion with superficial bulging in the gastric body was found during endoscopy and resembled the early gastric cancer. The final diagnosis was isolated gastric LCH. Nihei et al. (27) described a middle-aged female who exhibited atrophy and erosion of the gastric fundus mucosa that mimicked a signet ring cell carcinoma. The patient underwent a subtotal gastrectomy, and the postoperative pathological diagnosis confirmed isolated gastric LCH. Moreover, there was also a case of gastric LCH presenting as granulomatous lesions (28) in literature, thus highlighting the importance of differential diagnosis from other granulomatous diseases in stomach. Given the non-specific endoscopic manifestations, clinicians should carefully evaluate the diverse pathological morphology to avoid missed or incorrect diagnoses. In our case, gastroscopy revealed an erosive lesion on the greater curvature of the gastric antrum, suggesting chronic atrophic gastritis with erosion. The local hospital initially suspected a poorly differentiated carcinoma, but consultation with the pathology department of our hospital ascertained as isolated gastric LCH.

The diagnosis of LCH primarily relies on the histopathology and immunohistochemistry of the lesioned tissue. In isolated gastric LCH, the cytological morphology under light microscopy and immunohistochemical features generally correspond to those of LCH affecting other organs. Langerhans cells are relatively large and exhibit round or oval shapes. Their nuclei are irregular and are like kidney-shaped or coffee bean-shaped, with folded nuclear membranes and indistinct nucleoli. There is a prominent infiltration of reactive inflammatory cells in the background, such as neutrophils, eosinophils, lymphocytes, and multinucleated giant cells, etc. (29). For immunohistochemical characteristics, tumor cells specifically express langerin, CD1a, and S-100 (30). Notably, studies have reported Cyclin D1 overexpression in 30%–70% of LCH cases (31, 32), which may be attributed to the activation of RAS-RAF-MEK-ERK pathway (33). The histological findings showed typical cell morphology of LCH (**Figures 1A, B**) and immunohistochemistry testified the positivity of the genes mentioned above in this patient (**Figures 1C–H**), corresponding to the previous literature reports. It is worth noting that an atypical case of gastric LCH has also been documented. The case described an endoscopic spherical mass on the lesser curvature of the gastric body (34). Microscopic examination revealed Langerhans cells with significant atypia, obvious nucleoli, and easily visible mitotic figures. Despite these atypical morphological features, immunohistochemistry conformed to the LCH phenotype, showing strong positivity to S-100, CD1a, and langerin. The patient experienced a rapid disease progression and died two months after tumor resection. The cell morphology was highly malignant under the microscope, which penetrated into the perigastric lymph nodes and other organs around. The progression of disease was rapid, which was different from the conventional features of LCH. Therefore, for atypical cases with high-grade cytological atypia and aggressive behavior, we should be alert to the possibility of malignant transformation from LCH into Langerhans cell sarcoma (LCS). In such scenarios, the expression of CD56 plays a critical role in differential diagnosis. Although both LCH and LCS typically express S-100, CD1a, and langerin; CD56 positivity is highly suggestive of LCS and generally absent in classical LCH. This case displays CD56 negativity (**Figure 1I**) without the atypical cellular morphological features like LCS patients.

Furthermore, gastric LCH must be differentiated from other histiocytic diseases (35), for example, LCS, Rosai-Dorfman disease (RDD), and Erdheim-Chester disease (ECD); as well as non-histiocytic tumors (7), including poorly differentiated carcinoma, malignant melanoma, and mastocytosis, etc.

According to the literature, BRAF V600E mutation rate in isolated gastric LCH ranges from 66.7% to 90.9% (7, 36), which is significantly higher than that of LCH in other sites. To our knowledge, another few LCH-related mutations, such as MAP2K1, ARAF, and NRAS mutations, have not been reported in isolated gastric LCH. Those patients may have a different gene mutation spectrum by contrast other sites of LCH, warranting further investigation. Another study has suggested that the mutation rate of BRAF V600E was associated with the multi-system involvement and a poor prognosis in pediatric LCH (37). Interestingly, we find that BRAF V600E mutation rate is high in isolated gastric LCH among adults, however, the clinical prognosis generally appears favorable. The apparent discrepancy between the high mutation frequency and favorable prognosis may be explained by several factors. First, the BRAF V600E mutation itself does not uniformly predict aggressive behavior; rather, clinical outcomes depend on disease extent and organ involvement. Second, and most importantly, the cellular context in which the BRAF V600E mutation arises—rather than the mere presence of the mutation itself—appears to be the key determinant of clinical behavior. In the landmark study by Berres et al (38), the mutation was detected in circulating CD11c+ dendritic cells, CD14+ monocytes, and BM CD34+hematopoietic progenitors in all high-risk LCH patients, whereas it was restricted to lesional CD207+ DCs in low-risk patients. These observations support a model in which high-risk LCH arises from somatic mutation of a hematopoietic progenitor, while low-risk disease results from mutation of a tissue-restricted precursor DC. Therefore, in the case of isolated gastric LCH—which by definition is unifocal and single-system—the disease is likely derived from a tissue-restricted precursor rather than a multipotent hematopoietic stem cell, explaining its excellent prognosis despite a high BRAF V600E mutation rate. Third, the small number of reported cases limits the ability to draw definitive conclusions. Further research is needed to clarify the discrepancy between the high mutation rate and clinical prognosis in gastric LCH. Combined with this patient, BRAF V600E was positive in the local tissue by immunohistochemistry, but BRAF V600E mutation test was not performed, due to the limited specimen of pathological tissue. Regarding the impact on interpretation of the BRAF testing, Acosta-Medina et al. (39) has demonstrated that IHC has a sensitivity of 82.4% and a specificity of 96.4% compared with NGS as the gold standard. Given the high specificity of IHC, we consider the positive result sufficient for clinical purposes in our case. Nevertheless, we acknowledge that molecular testing should be pursued whenever tissue is available.

Isolated gastric LCH most commonly presents as a solitary polypoid lesion (40, 41). For a unifocal lesion, the treatment mainly involves observation or endoscopic resection of the lesion, while multifocal lesions are recommended chemotherapy or targeted therapy. The 5-year overall survival rate exceeds 90% for adult patients with single-system LCH (42, 43). Existing data indicate that isolated gastric LCH has a favorable prognosis similar to single-system LCH at other sites. A study of 13 cases of gastrointestinal LCH (including 8 gastric cases) found that endoscopic morphology correlated with patients’ prognosis. The progression-free survival rate of submucosal protruding lesions was significantly better than that of non-protruding lesions (22). These endoscopic characteristics may provide a basis for guiding treatment and predicting prognosis. Based on these findings, we speculate that close follow-up observation is sufficient for polyps or submucosal protruding lesions under endoscopy, while ulcerative lesions might require the earlier intervention. Although this requires validation in large cohorts. Kim et al. (20) studied seven patients with gastric LCH presenting as superficial erosions (diameter < 0.5cm) or protruding lesions with erosion under endoscopy. Only one patient underwent endoscopic resection, while the other six patients were managed with regular surveillance and eventually showed spontaneous regression of the disease. Accordingly, they proposed that careful periodic monitoring may be sufficient for isolated gastric LCH. Most importantly, the International Expert Consensus Recommendations (43) state that for unifocal LCH, “observation or local therapies such as surgical excision” are both acceptable first-line options. This patient had a single 0.5 cm × 0.5 cm erosive lesion on the gastric antrum mucosa. Taken together, we recommend close surveillance, with endoscopic resection reserved for cases of progression. For unifocal disease, initial follow-up imaging is recommended every 3–6 months, and surveillance intervals may be extended once disease stability is confirmed. Our patient has been kept under close surveillance by the gastroenterology team. One year after diagnosis (March 2026), repeated endoscopy confirmed a stable lesion with no evidence of progression. The patient’s gastrointestinal symptoms have resolved following symptomatic management then.

## 4 Conclusion

Isolated gastric LCH in adults is making it prone to miss diagnosis and misdiagnosis due to its extremely low incidence and lack of specific clinical manifestations. In clinical practices, if erosive or ulcerative lesions are found under gastroscopy, which shows a large amount of inflammatory cells infiltration in pathology, and which are ineffective against standard therapy, the possibility of LCH should be considered. For atypical cases with obvious cellular atypia, LCS must be ruled out. BRAF V600E mutation appears more prevalent in isolated gastric LCH compared to LCH involving other organs, however, larger-scale studies are required to elucidate its molecular pathological changes more comprehensively. For single-system unifocal patients without symptoms, it is recommended active surveillance or endoscopic resection of the lesion; whereas patients with multi-system multifocal involvement should receive chemotherapy or targeted therapy. The overall prognosis of isolated gastric LCH is generally favorable. Notably, the progression-free survival rate for endoscopic submucosal protruding lesions is better than that of non-protruding lesions, which may indicate a correlation with clinical prognosis and merits further investigation in larger cohorts.

## Data Availability

The datasets used and analyzed during the current study are available from the corresponding author request.
